# Unveiling Breakthroughs in Post-resuscitation Supportive Care for Out-of-Hospital Cardiac Arrest Survivors: A Narrative Review

**DOI:** 10.7759/cureus.44783

**Published:** 2023-09-06

**Authors:** Nikhil Sai Jagarlamudi, Kriti Soni, Saima S Ahmed, Naga Sai Ram Makkapati, Sujaritha Janarthanam, Cristhian R Vallejo-Zambrano, Khushbu C Patel, Roshni Xavier, Praveen Kumar Ponnada, Iqra Zaheen, Muhammad Ehsan

**Affiliations:** 1 Internal Medicine, NRI Medical College, Guntur, IND; 2 Internal Medicine, Dr. D. Y. Patil Medical College, Hospital & Research Center, Pune, IND; 3 Internal Medicine, Dow International Medical College, Karachi, PAK; 4 Internal Medicine, NRI Medical College Guntur, Vijayawada, IND; 5 Internal Medicine, Sri Ramachandra Institute of Higher Education and Research Center, Chennai, IND; 6 General Practice, Colegio de Médicos de Manabí, Manta, ECU; 7 Internal Medicine, Medical College Baroda, Baroda, IND; 8 Internal Medicine, Rajagiri Hospital, Aluva, IND; 9 Internal Medicine, Carewell Hospital, Malappuram, IND; 10 Internal Medicine, Apollo Institute of Medical Science and Research, Hyderabad, IND; 11 Internal Medicine, Jinnah Medical and Dental College, Karachi, PAK; 12 General Medicine, International Medical Graduates (IMG) Helping Hands, Lahore, PAK

**Keywords:** post-resuscitation, post-resuscitation care, hemodynamic support, ecmo, targeted temperature management, cardiac arrest

## Abstract

Survivors of out-of-hospital cardiac arrest (OHCA) experience significant mortality rates and neurological impairment, potentially attributed to the hypoxic-ischemic injury sustained amid the cardiac arrest episode. Post-resuscitation care plays a crucial role in determining outcomes for survivors of OHCA. Supportive therapies have proven to be influential in shaping these outcomes. However, targeting higher blood pressure or oxygen levels during the post-resuscitative phase has not been shown to offer any mortality or neurological benefits. In terms of maintaining hemodynamic instability after resuscitation, it is recommended to use norepinephrine rather than epinephrine. While extracorporeal cardiopulmonary resuscitation has shown promising results, targeted temperature management has been found ineffective in improving outcomes despite its previous potential. This review also investigates various challenges and barriers associated with the practical implementation of these supportive therapies in clinical settings. The review also highlights areas ripe for future research and proposes potential directions to further enhance post-resuscitation supportive care for OHCA survivors.

## Introduction and background

An out-of-hospital cardiac arrest (OHCA) refers to an abrupt stoppage of mechanical heart activity outside a hospital environment, which is confirmed by the lack of signs of circulation [1]. OHCA is a significant cause of cardiac arrests in Europe and the United States, with approximately 356,000 cardiac arrests annually in the United States [2] and approximately 275,000 in Europe [3]. Despite advancements in resuscitative efforts, survivors of OHCA continue to face substantial risks of morbidity and mortality [4].

Neurons are very susceptible to hypoxic-ischemic insults and are therefore easily damaged due to hypoperfusion thus neuronal injury is the major cause of mortality and morbidity in OHCA survivors [4], followed by cardiac instability due to arrhythmias [5]. The presence of other factors, such as non-shockable rhythm, longer time between arrest and CPR, and lower ejection fraction at the time of discharge, is also linked with long-term morbidity and mortality in OHCA survivors [4]. Post-resuscitation care has emerged as a pivotal determinant in shaping outcomes for OHCA survivors, transcending the acute phase of intervention. Supportive management like the use of targeted temperature management (TTM), glycemic control, neuroprotective drugs, ventilatory support, and maintaining normocapnia in comatose patients have shown to improve the long-term outcomes in patients with OHCA, thus integrating these principles into post-resuscitation care guidelines [6].

Evidence indicates that TTM has the potential to reduce brain damage and improve neurological outcomes. It is recommended especially in comatose OHCA adults who remained unresponsive after the return of spontaneous circulation (ROSC) [6]. Cardiac rehabilitation has shown evidence to reduce mortality, improve quality of life, and cost-effectiveness [6]. Maintaining normocapnia in comatose OHCA survivors has improved neurological outcomes; however, the evidence is not conclusive [7]. Identifying and treating underlying adverse events like seizures with appropriate antiepileptic medications have shown overall improvements [6]. Although there is research suggesting that supportive management during post-resuscitation care can be effective, there is not enough evidence to implement it in clinical practice due to conflicting results of various studies. In light of recent trials [7-11] evaluating various supportive therapies for post-resuscitation care of OHCA survivors, there is a need for a comprehensive narrative review to summarize all the available evidence regarding supportive care of OHCA survivors.

This review seeks to offer a comprehensive examination of the present condition of supportive care following successful resuscitation for individuals who OHCA. The review focuses on recent breakthroughs and advancements in post-resuscitation care tailored to OHCA survivors, analyzing their impact on survival rates, functional outcomes, and overall quality of life. Additionally, it examines challenges and barriers associated with the practical implementation of these breakthroughs in clinical settings. The review also highlights areas ripe for future research and proposes potential directions to further enhance post-resuscitation supportive care for OHCA survivors, contributing to the refinement of clinical practices and ultimately improving patient outcomes in this critical field.

## Review

Methodology

Various databases such as MEDLINE (via Pubmed), Cochrane Library, and Embase were searched to retrieve relevant studies. The literature search used terms related to “cardiac arrest,” “targeted temperature management,” “supportive management,” and “post-resuscitation.” Two authors independently screened the search results and any conflict among them was resolved by the third author.

Targeted temperature management (TTM)

TTM, or protective hypothermia, can reduce cerebral metabolic rate after resuscitation by lowering the demand for cerebral oxygen [12]. By reducing the body's core temperature, TTM helps to minimize the deleterious effects of ischemia-reperfusion injury, inflammation, and excitotoxicity that can occur after a cardiac arrest [13]. Furthermore, it has been shown to decrease metabolic rate, reduce oxidative stress, and limit the release of harmful neurochemicals, all of which can contribute to improved neurological function and overall patient outcomes [14]. Hypothermia is commonly employed to protect the brain from ischemic damage during cardiac surgery involving cardiopulmonary bypass, as well as in neurosurgical procedures. Additionally, ongoing research is exploring its potential application as a therapeutic approach for conditions like ischemic stroke and traumatic brain injury [15].

Previously, many clinical trials have shown that TTM can significantly improve neurological outcomes and overall survival rates in post-resuscitation care in patients who have suffered OHCA [16,17]. However, recent trials and systematic reviews did not demonstrate its effectiveness in post-resuscitative care.

A meta-analysis of seven randomized controlled trials by Aneman et al. aimed to assess the impact of TTM in post-resuscitation care [18]. They found no significant decrease in mortality rates (RR 0.95; 95%CI 0.78 to 1.09) and a decrease in unfavorable neurological outcomes (RR 0.93; 95%CI 0.84 to 1.02) with the use of TTM [18]. Another meta-analysis found that the use of TTM confers no survival benefit (RR 1.06; 95%CI 0.94 to 1.20) in OHCA survivors as compared to normothermia [19]. Incidence of arrhythmias was, however, seen to be increased in the TTM group (RR 1.35; 95%CI 1.16 to 1.57) [19]. In a trial by Dankiewicz et al., 1,900 comatose cardiac arrest survivors were assigned to undergo TTM at 33°C or targeted normothermia [11]. Mortality rates were found to be similar between the two groups (RR 1.04; 95%CI 0.94 to 1.14) at six months. The risk of arrhythmia was increased in the TTM group [11].

The latest guideline advocates the optimization of TTM during the post-resuscitation phase to improve outcomes and long-term neurological rehabilitation [20]. Following cardiac arrest in comatose patients, it recommends active prevention of fever for at least 72 hours. However, the latest trial evaluating the efficacy of active fever prevention did not find any difference in mortality or disability between the 72-hour or 36-hour group [9].

In conclusion, while clinical guidelines advocate for the use of TTM, its suboptimal performance in recent trials beckons for a profound re-evaluation and prompts the need for large-scale randomized controlled trials to determine its role in cardiac arrest survivors.

Hemodynamic optimization

Hemodynamic instability often follows successful resuscitation from OHCA, arising due to the complex interplay of various pathophysiological mechanisms such as reperfusion injury, myocardial dysfunction, and systemic inflammatory response syndrome (SIRS) [21]. This can lead to cerebral hypoperfusion, which in turn can result in poor neurological outcomes and increased mortality rates in post-resuscitation patients [22]. Hence, an elevated mean arterial blood pressure (MAP) following resuscitation from a cardiac arrest might enhance blood flow in the brain by increasing cerebral perfusion pressure, which could potentially reduce ongoing cerebral damage [23].

Guidelines advocate for keeping MAP equal to or greater than 65 mm Hg in the post-resuscitation period [6,24]. The latest BOX trial evaluated the effect of a high (77 mm Hg) and a low (63 mm Hg) blood pressure target on morbidity and mortality in OHCA survivors. No difference was observed regarding mortality or severe disability between the two groups [8].

A recent meta-analysis by Cheema et al. did not find any significant difference regarding mortality between higher versus lower BP target goals (OR 1.12; 95%CI 0.86 to 1.45) in post-OHCA patients [25]. Both groups have a similar incidence of arrhythmias and neurologic outcomes, but the duration of ICU stay was shorter among those in the higher BP target category [25].

Fluid resuscitation is the initial intervention to restore intravascular volume and improve cardiac output [26]. Hemodynamic support can be achieved through the use of vasoactive medications such as norepinephrine or epinephrine when hemodynamic objectives are not met even after optimizing preload [27]. Bougoin et al. conducted an observational study to assess the role of different vasodilators in post-resuscitation shock [28]. The study advocated for the use of norepinephrine in the post-resuscitative period as epinephrine was linked with higher rates of overall mortality (OR 2.6; 95%CI 1.4-4.7) and cardiovascular-related hospital mortality (OR 5.5; 95%CI 3.0-10.3) [28].

Ventilation targets

Oxygen Targets

Hypoxic ischemic encephalopathy (HIE) stands as the predominant cause of impairment and fatality subsequent to OHCA. It is common for patients to experience hypoxic brain damage upon the restoration of spontaneous circulation [29]. More than 80% of cardiac arrest survivors who are admitted after resuscitation in the ICU are comatose and of them, 66% of patients have a devastating outcome due to hypoxic brain injury [30]. Earlier investigations have hinted at a decline in cerebral blood flow and a surge in oxygen demand among cardiac arrest patients, exacerbating HIE [31,32]. This highlights the importance of providing oxygen therapy to post-cardiac arrest patients.

Although oxygen is commonly administered as a treatment for individuals revived from OHCA, there exists limited evidence to provide healthcare practitioners with definitive guidance on the optimal utilization of oxygen for this particular patient group [33]. An excessive amount of oxygen in the bloodstream can potentially have negative effects during the reperfusion phase after cardiopulmonary resuscitation (CPR). A recent cohort study conducted by Roberts et al. suggests that immediate hyperoxia following cardiac arrest can lead to poor neurological function upon hospital discharge (RR 1.23; 95%CI 1.11-1.35) [34].

In a study by Kuisma et al., individuals who were successfully resuscitated after an OHCA were divided into two groups [35]. These groups were randomly assigned to receive ventilation with either 30% or 100% oxygen for 60 minutes after the ROSC. The results of this study suggest that the group receiving 100% oxygen experienced superior outcomes compared to the group receiving only 30% oxygen [35].

According to a meta-analysis conducted by Young et al., conservative oxygen therapy, as opposed to liberal oxygen therapy, led to a significant decrease in mortality (OR 0.58; 95%CI 0.35 to 0.96) [36]. However, it must be noted that the certainty of these findings was somewhat limited due to bias and imprecision [36]. However, a more recent meta-analysis by Cheema et al. which included nine RCTs found no significant difference in all-cause mortality between the conservative and liberal oxygen target groups (RR 0.95 95%CI 0.80 to 1.13) [31].

Based on a comprehensive review of multiple studies conducted over time, the current guidelines from ERC-ESICM for post-resuscitation care recommend maintaining pulse oximetry levels within a designated “safe range” of 94%-98%. This approach is advocated to prevent both hypoxia and hyperoxia [6].

CO_2_ Targets

Patients who suffer from cardiac arrest frequently experience high PaCO_2_ after ROSC, which typically returns to baseline in an hour. PaCO_2_ is an important regulator of cerebral blood flow. After a cardiac arrest, CO_2_ derangements can have detrimental effects, necessitating careful CO_2_ monitoring in comatose patients during the post-resuscitation period after ROSC for improved neurological outcomes [37]. Current guidelines state that the target PaCO_2_ should be in the range of 4.5-6.0 kPa or 35-45 mm Hg in patients requiring mechanical ventilation after ROSC [6].

Moderate hypercapnia improves brain oxygenation, minimizing damage caused by ischemia-reperfusion injury [38]. A prospective cohort study done by Vaheersalo et al. showed that moderate hypercapnia is associated with improved outcomes (OR=1.015; 95%CI 1.002 to 1.029) [39]. However, a recent RCT done by Eastwood investigating the effects of targeted mild hypercapnia and normocapnia did not show any significant difference in neurological outcomes between the two cohorts (RR=0.98; 95%CI 0.87 to 1.11) [7].

In a retrospective cohort study, Okada et al. aimed to understand the relationship between carbon dioxide levels and neurological function after one month in OHCA patients [40]. They found that maintaining normal carbon dioxide levels and slightly higher carbon dioxide levels during the first 24 hours following the ROSC was linked with improved neurological outcomes, as opposed to having very low or high carbon dioxide levels. Hypercapnia after ROSC was also seen to be associated with a longer duration of CPR, poor neurological outcomes, and a high mortality rate [40].

Observational studies have indicated that survivors of OHCA who undergo TTM are susceptible to developing hypocapnia, a condition that can be averted by monitoring CO_2_ levels through arterial blood gas (ABG) analysis and end-tidal CO_2_ monitoring [41].

Glycemic targets

Derangements in glucose levels following cardiac arrest are common in diabetic and non-diabetic patients [42]. The duration of cardiac arrest can influence the mechanisms responsible for maintaining glucose balance, with prolonged arrest durations resulting in more pronounced ischemia-reperfusion (I/R) injury, heightened oxidative stress, inflammation, and disruption of glucose regulation [43]. Elevated blood glucose levels following ROSC are associated with heightened rates of mortality and neurological impairment [44].

The European Resuscitation Council proposes that blood glucose levels should be kept above 180 mg/dL, and measures should be taken to prevent hypoglycemia [6]. A study by Zhou et al. found that prolonged time spent in the blood glucose range of 70 to 140 mg/dL is closely linked to an increased rate of hospital survival among patients who experienced pre-hospital cardiac arrest (PCA) [45]. Elevated blood sugar levels (> 180 mg/dL) are prevalent in PCA patients and are correlated with an increased risk of mortality [45].

Evidence regarding glucose control in the post-resuscitation period highlights the need for more trials to evaluate the most optimal glucose management strategies after resuscitation in OHCA survivors.

Extracorporeal life support (ECLS) in post-resuscitation care

ECLS is a highly advanced medical procedure designed to offer temporary mechanical support to patients who have experienced cardiac arrest that is refractory to traditional CPR [46]. By temporarily diverting blood outside the body for oxygenation and carbon dioxide removal, ECLS serves as an adjunctive therapy to conventional resuscitative measures. ECLS plays a critical role in restoring hemodynamic stability and oxygenation, particularly in cases of refractory cardiac arrest and profound shock, thus augmenting the chances of successful post-resuscitation recovery [47].

A meta-analysis by Alfalasi et al. studied the impact of extracorporeal CPR (ECPR) compared to traditional CPR in 18,620 patients with OHCA [48]. Patients who underwent ECPR achieved favorable neurologic outcomes at both three and six months when compared with CPR. However, no significant survival benefit was established [48]. Another systematic review also established the beneficial role of ECPR in patients with OHCA [49]. However, the latest trial by Suverein et al. did not find any significant mortality benefit in patients who underwent ECPR during refractory cardiac arrest (OR 1.4; 95%CI 0.5 to 3.5) [10].

Seizure control

Seizures manifest in OHCA patients as a result of the hypoxic-ischemic brain injury sustained during the cardiac arrest episode [50]. Seizures can have detrimental effects on the recovery and prognosis of OHCA survivors, leading to additional neurological injury and impairments [51]. The prognostic significance of seizures remains unaffected by the level of the target temperature [52]. The latest evidence suggests that non-convulsive status epilepticus (NCSE) has a better prognosis when compared to convulsive seizures in post-resuscitated patients [53].

Anticonvulsants' role in OHCA patients has not been extensively researched. Kim et al. evaluated the impact of anticonvulsants on 930 patients who have survived OHCA [54]. They found anticonvulsant use to be associated with poor neurological outcomes (aOR 1.69; 95%CI 1.03 to 2.77) [54].

Additional research is necessary to gain deeper insights into the impact of anticonvulsant drugs on individuals who have survived OHCA. Additionally, it could help establish guidelines regarding the use of these drugs in clinical practice when caring for OHCA survivors.

Role of antibiotics

Infections frequently complicate recovery and increase ICU stays for OHCA survivors. More than half of survivors suffer from pneumonia, and around 10% develop sepsis [55]. These patients had increased hemodynamic instability and significantly higher short-term mortality [56]. However, no significant association between hospital mortality and sepsis is observed in OHCA patients who survived their ED stay (OR 1.3; 95%CI 0.2 to 9.2) in a study by Dagher et al. [57].

Although beneficial, therapeutic hypothermia (TH) can impair the immune response, increasing the risk of infections. TH further challenges the situation by masking pyrexia [58]. A retrospective study by Binks and Thomas on patients treated with TH revealed a 29% infection rate, raising the possibility of infection incidence in this population [59]. The study further concluded a positive association between an increased infection rate and the length of an ICU stay [59]. Another retrospective study by Hellenkamp et al. revealed a higher incidence of early pneumonia in OCHA patients who received TH [58]. This study also demonstrated that patients who received delayed antibiotic therapy spent longer in the hospital and the ICU [58].

A meta-analysis by Couper et al. compared the effects of early antibiotic initiation and delayed or clinically driven antibiotics in survivors [60]. The results showed no significant benefit of early initiation in increasing survival outcomes (OR 1.16; 95%CI 0.97 to 1.40) [60]. In contrast, a study done by Davies et al. showed a significant reduction in mortality in patients who had early initiation of antibiotics compared to patients who did not [61]. Several factors, like resistance to the chosen antibiotic and a longer time gap between studies, might have contributed to these conflicting outcomes [61]. Moreover, results can be confounded by a higher number of variables in observational studies. These limitations necessitate the need for extensive research in this area.

Patients who were administered prophylactic antibiotics showed a markedly reduced incidence of pneumonia (12.6% versus 54.9%) and sepsis (1.2% versus 5.7%) in comparison to those who did not [62]. One hundred ninety-eight patients who were admitted to the ICU following OHCA participated in a randomized control trial carried out by François et al. [63]. Patients who received prophylactic amoxicillin-clavulanic acid had a reduced occurrence of early VAP when compared to those in the placebo group (HR 0.53; 95%CI 0.31 to 0.92). Nonetheless, there was no significant benefit observed concerning late-onset VAP, length of hospital stays, or the number of days without ventilation support [63].

## Conclusions

Despite the advances in resuscitative efforts, individuals who survive OHCA are still faced with significant risks of morbidity and mortality. To improve outcomes for these survivors, several supportive therapies have been explored, including TTM, aggressive hemodynamic control, maintenance of specific ventilation targets, control of glucose derangements and seizures, treatment of infections, and the use of ECPR. These strategies have shown promise in various trials and are now integrated into post-resuscitation care guidelines.

Recent clinical trials have produced conflicting results on the effectiveness of certain therapeutic interventions. TTM, higher blood pressure, and oxygen targets did not demonstrate significant benefits in terms of mortality or morbidity for OHCA survivors. Further research is needed to clarify their role in improving patient outcomes after OHCA. Ongoing studies may also require revisions to existing guidelines. It is crucial to optimize the use and implementation of these supportive therapies in order to enhance post-resuscitation care for OHCA survivors and ultimately improve their long-term outcomes and quality of life.
